# Loss of skeletal muscle mass during neoadjuvant treatments correlates with worse prognosis in esophageal cancer: a retrospective cohort study

**DOI:** 10.1186/s12957-018-1327-4

**Published:** 2018-02-12

**Authors:** Tommi Järvinen, Ilkka Ilonen, Juha Kauppi, Jarmo Salo, Jari Räsänen

**Affiliations:** 1 Department of General Thoracic and Esophageal Surgery, Heart and Lung Center, Helsinki University Hospital, P.O. Box 340 HUS, FIN-00029 Helsinki, Finland; 2 Department of Surgery, Clinicum, University of Helsinki, Helsinki, Finland

**Keywords:** Esophageal neoplasms, Thoracic surgery, Sarcopenia, Body composition, Malnutrition

## Abstract

**Background:**

Nutritional deficits, cachexia, and sarcopenia are extremely common in esophageal cancer. The aim of this article was to assess the effect of loss of skeletal muscle mass during neoadjuvant treatment on the prognosis of esophageal cancer patients.

**Methods:**

Esophageal cancer patients (*N* = 115) undergoing neoadjuvant therapy and surgery between 2010 and 2014 were identified from our surgery database and retrospectively analyzed. Computed tomography imaging of the total cross-sectional muscle tissue measured at the third lumbar level defined the skeletal muscle index, which defined sarcopenia (SMI < 52.4 cm2/m2 for men and < 38.5 cm2/m2 for women). Images were collected before and after neoadjuvant treatments.

**Results:**

Sarcopenia in preoperative imaging was prevalent in 92 patients (80%). Median overall survival was 900 days (interquartile range 334–1447) with no difference between sarcopenic (median = 900) and non-sarcopenic (median = 914) groups (*p* = 0.872). Complication rates did not differ (26.1% vs 32.6%, *p* = 0.725). A 2.98% decrease in skeletal muscle index during neoadjuvant treatment correlated with poor 2-year survival (log-rank *p* = 0.04).

**Conclusion:**

Loss of skeletal muscle tissue during neoadjuvant treatment correlates with worse overall survival.

**Electronic supplementary material:**

The online version of this article (10.1186/s12957-018-1327-4) contains supplementary material, which is available to authorized users.

## Background

Esophageal cancer (EC) is intimately related to weight changes and poor nutritional status, since the most common symptoms of EC are dysphagia and weight loss [1]. In locally advanced EC, baseline nutritional status has been linked to survival after definitive chemoradiotherapy [2]. Preoperative weight loss has also been linked to worse outcomes [3]. A high body mass index (BMI) has not been found to have a significant effect on survival in EC [4, 5].

In the recent years, there has been an increasing amount of studies on frailty and especially sarcopenia as prognostic factors in cancers. Sarcopenia is defined as the progressive loss of muscle related to aging or disease [6]. Sarcopenia has been associated with worse outcomes in many types of cancers such as hepatocellular carcinoma, colorectal cancer, and small cell lung cancer [7–9]. For esophageal and gastroesophageal junction cancers, there are conflicting reports. Worse long-term outcomes have been reported in resected esophageal or gastroesophageal junctional cancers [10–12]. Sarcopenia has been linked to increased pulmonary and other complication rates [13, 14]. Decreased skeletal muscle area during neoadjuvant therapy has also been associated with poorer outcomes and risk of positive clinical resection margin [15, 16]. There are also reports of sarcopenia not being an independent prognostic risk factor for mortality, morbidity, or poor outcomes in EC after neoadjuvant chemoradiotherapy or chemotherapy [14, 17–19]. One study found a significant correlation with lean psoas mass and survival in patients not undergoing neoadjuvant treatment, but no such effect on patients receiving neoadjuvant treatment [20].

The aim of this trial was to assess the effect of sarcopenia and loss of skeletal muscle index during neoadjuvant treatments in patients undergoing esophagectomy for EC. The primary end-point is overall survival, and secondary end points are recurrence-free survival and complication rates.

## Methods

### Patients

Patients who underwent surgical resection and neoadjuvant therapy for EC between 2010 and 2014 were identified in retrospect from our surgery database using type of surgery and diagnosis of esophageal or junctional cancer as identifiers (*N* = 118). Patients who had no eligible imaging for analysis of the skeletal muscle index were excluded (*N* = 3), leaving 115 patients. Collected data included patient characteristics, weight, and weight-loss data before and during the treatment and follow up, primary tumor characteristics and staging, specifics of neoadjuvant, endoscopic, surgical and adjuvant treatments, post-operative and long-term complications, and overall survival. Weight loss here is defined as unintentional deviation from healthy weight (weight 6 months before diagnosis). Complications were collected as recommended by an international consensus statement [21]. 30- and 90-day overall survival rates and 2-year overall survival and recurrence-free survival rates were also collected.

CT (computed tomography) scans were collected from time of initial staging, post-neodjuvant, 6-, and 18-month follow-up visits. CT scans were excluded if there was impaired visibility at third lumbar vertebra or over 1 month of time interval between the CT scan and associated event (start of neoadjuvant therapy, operation, 6-month follow-up visit, or 18-month follow-up visit).

### Preoperative staging

All patients underwent gastroscopies with biopsies that confirmed the diagnosis of EC. All patients underwent CT scans of the thorax, abdomen, and pelvis and a routine total body PET-CT scan. Endoscopic ultrasound was done unless prevented by tumor obstruction or stent insertion and assessed the invasion depth of the tumor and identified regional enlarged lymph nodes. PET/CT scans were routinely repeated after neoadjuvant treatments for preoperative planning and to assess the radiologic response of the tumor.

### Neoadjuvant treatment protocols

Treatment strategies were discussed and decided together with oncologists. Patients with nodal disease spread (cN+) or transmural tumor invasion (cT ≥ 3) underwent neoadjuvant treatment, unless contraindicated. Neoadjuvant therapy was epirubicin–oxaliplatin–capecitabine neoadjuvant chemotherapy as per MAGIC (Medical Research Council Adjuvant Gastric Infusional Chemotherapy) protocol for esophageal adenocarcinoma [22]. Squamous cell carcinoma was treated with neoadjuvant chemoradiotherapy consisting of 2 cycles of platin- and 5-fluorouracil-based therapy over 5–6 weeks. Chemosensitization was followed by a 45 Gy total dose of radiation to the tumor and regional nodes, in 1.8 Gy daily fraction.

### Preoperative endoscopic procedures

Endoscopic mucosal resection (EMR) with or without radiofrequency ablation (RFA) was discussed as an option for patients with mucosal tumors or high-grade dysplasia. The initial decision whether to proceed with endoscopic treatments or to do an esophagectomy was a shared decision between the surgeon and the patient. If endoscopic mucosal resection showed submucosal spread or there was cancer recurrence, treatment proceeded to esophagectomy. One patient included in this study had a prior EMR. Patients with obstructing tumor growth and marked dysphagia preventing the ingestion of solid foods were treated with insertion of a self-expandable metallic stent (SEMS, *N* = 35) or a percutaneous endoscopic gastrostomy (PEG, *N* = 4) tube.

### Surgical treatment and follow-up

Surgical techniques included minimally invasive esophagectomy, hybrid minimally invasive esophagectomy with either laparoscopy or video-assisted thoracoscopic surgery, Ivor-Lewis esophagectomy, 3-part esophagectomy, and transhiatal esophagectomy. These techniques are described elsewhere [23]. Post-operative stage of the tumor was reported according to eighth edition AJCC/IAUC staging [24]. Amount of positive lymph nodes and total lymph node count was recorded.

Patients were followed until death or January 2017, yielding a follow-up period of at least 24 months. Patients were met at an outpatient clinic 1 month after surgery to assess the recovery from surgery. Gastroscopies were done every 6 months for 2 years after surgery and annually for up to 5 years. CT scans were taken 6 months after surgery, 18 months after surgery, and annually up to 5 years.

All the treatments discussed here are standard approaches in our institution.

### Measurement of muscle parameters and sarcopenia definition

Scans were coded in order to blind the researcher from outcome. Images were imported to Osirix® Version 3.3 (32-bit Pixmeo, Sarl, Switzerland). We selected a single image on the level of L3, with both transverse processes and delineated abdominal muscles by use of a semi-automated selection of region of interest. Psoas, quadratus lumborum, paraspinal, transverse abdominal, external oblique, internal oblique, and rectus abdominis muscles were included. The Hounsfield unit threshold range for skeletal muscle was − 29 to + 150. The images were manually corrected, if needed, by the propulsion and brush tools in Osirix©. The cross-sectional total muscle area at the level of L3 (cm2) was divided by the square of height (m2), which produced the skeletal muscle index (SMI). This method is suggested as the preferred method of measuring the muscle mass of cancer patients [25]. SMI limit for sarcopenia was < 52.4 cm2/m2 for men and < 38.5 cm2/m2 for women, based on a previous study by Prado et al. [26] For the survival and complication analyses, the preoperative SMI values were used, unless stated otherwise.

**Fig. 1:**
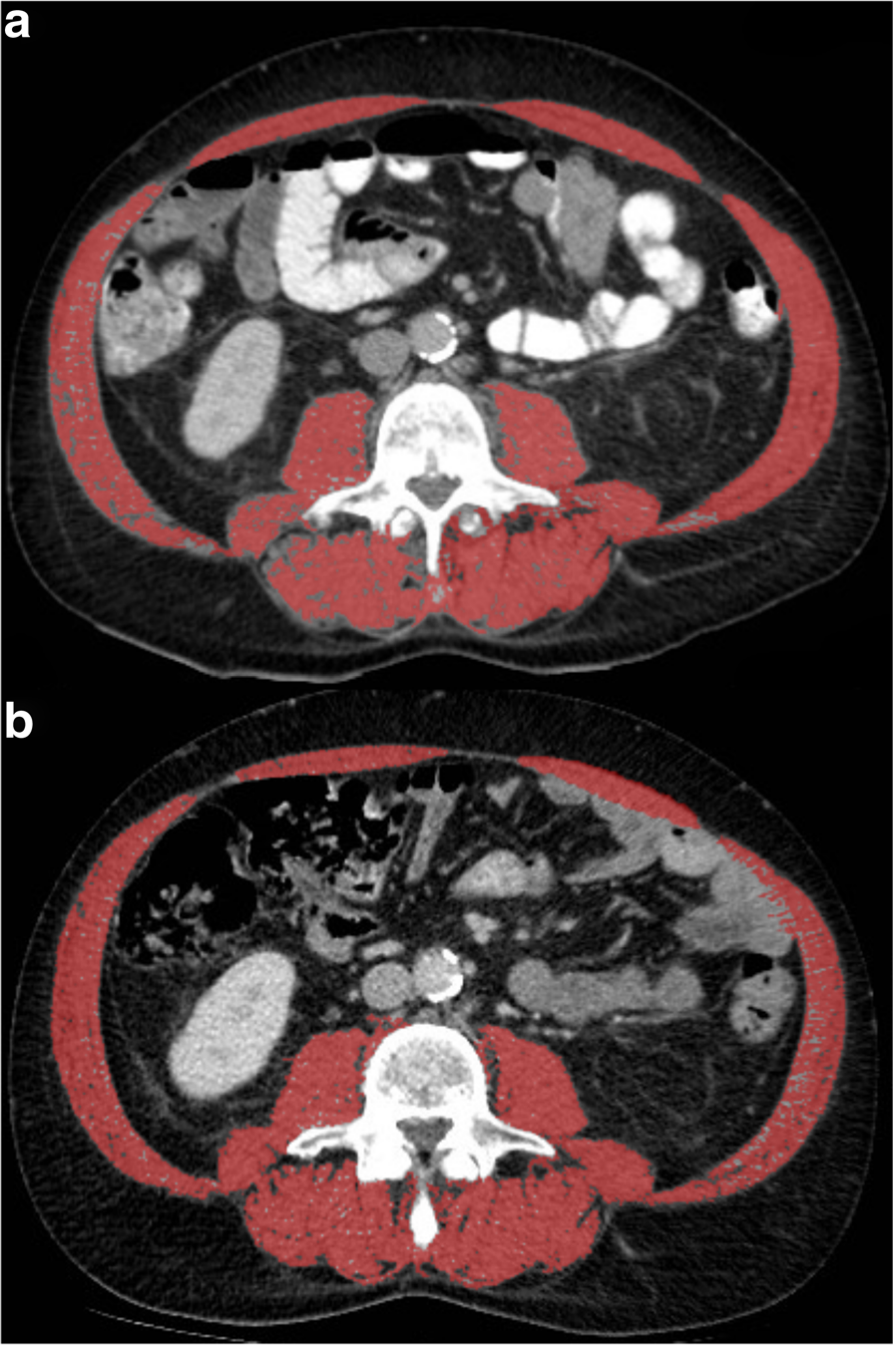
An example of SMI delineating. **a** Shows a male patient without sarcopenia (SMI = 57.6 cm2/m2), whereas **b** shows the same patient with sarcopenia (SMI = 47.9 cm2/m2) at follow-up

The process of delineating the abdominal muscle mass is shown in Fig. 1.

### Statistical analysis

All statistical analysis was done with R Project. (R Core Team, 2016). R: A language and environment for statistical computing. R Foundation for Statistical Computing, Vienna, Austria. URL: https://www.R-project.org/).

Continuous parameters were tested for normality using Shapiro-Wilk’s test and deemed normally distributed if *p* > 0.05. Normal continuous data is presented as mean and standard deviation (SD) whereas non-normal data is described with median and interquartile range (IQR). For comparing normal scalar variables between two groups, independent samples Student’s *t* test was used and for non-normal variables Mann-Whitney *U* test was used. The 2-tailed *χ*^2^ test served for categorical variables. Kaplan-Meier survival analysis and log-rank test demonstrated the possible difference of survival between groups.

## Results

Baseline characteristics of the study population are displayed in Table 1*.* Included patients numbered 115, of which sarcopenia was found in 92 (80%). Median overall survival was 900 days (interquartile range 334–1447). Patients with and without preoperative sarcopenia are compared in Table 1*.* The sarcopenic patients were statistically significantly older, taller, weighed less, and had smaller prevalence of current smokers.

**Table 1:** Baseline characteristics of the study patient population

	Overall		Pre-operative sarcopenia				*p*
			No		Yes		
Number of patients (%)	115		23		92		
Sex (%)							
Female	29	(25.2)	9	(39.1)	20	(21.7)	0.147
Male	86	(74.8)	14	(60.9)	72	(78.3)	
ECOG (%)							
0	40	(35.1)	11	(47.8)	29	(31.9)	0.140
1	69	(60.5)	10	(43.5)	59	(64.8)	
2	5	(4.4)	2	(8.7)	3	(3.3)	
Smoking status (%)							
Current smoker	32	(27.8)	11	(47.8)	21	(22.8)	0.018
Ex-smoker	40	(34.8)	3	(13.0)	37	(40.2)	
Non-smoker	43	(37.4)	9	(39.1)	34	(37.0)	
T stage (%)							
1	2	(1.8)	0	(0.0)	2	(2.2)	0.894
2	10	(8.9)	2	(8.7)	8	(9.0)	
3	86	(76.8)	17	(73.9)	69	(77.5)	
4	14	(12.5)	4	(17.4)	10	(11.2)	
N stage (%)							
0	33	(29.5)	6	(26.1)	27	(30.3)	0.258
1	74	(66.1)	15	(65.2)	59	(66.3)	
2	4	(3.6)	1	(4.3)	3	(3.4)	
3	1	(0.9)	1	(4.3)	0	(0.0)	
Cancer type (%)							
Adenocarcinoma	88	(76.5)	21	(91.3)	67	(72.8)	0.061
SCC^*^	27	(23.5)	2	(8.7)	25	(27.2)	
Pathological grade^**^ (%)							
1	9	(16.1)	2	(28.6)	7	(14.3)	0.723
2	22	(39.3)	3	(42.9)	19	(38.8)	
3	25	(44.7)	2	(28.6)	23	(46.9)	
Tumor location (%)							
Lower third	95	(82.6)	20	(87.0)	75	(81.5)	0.646
Middle third	17	(14.8)	3	(13.0)	14	(15.2)	
Upper third	3	(2.6)	0	(0.0)	3	(3.3)	
Neoadjuvant treatment (%)							
Chemoradiation	28	(24.3)	2	(8.7)	26	(28.3)	0.051
Chemotherapy	87	(75.7)	21	(91.3)	66	(71.7)	
Operation type (%)							
MIE^†^	78	(67.8)	18	(78.3)	60	(65.2)	0.464
Thoracotomy	26	(22.6)	2	(8.7)	24	(26.1)	
Hybrid-laparoscopy	5	(4.3)	1	(4.3)	4	(4.3)	
Hybrid-VATS^‡^	3	(2.6)	1	(4.3)	2	(2.2)	
Transhiatal	3	(2.6)	1	(4.3)	2	(2.2)	
Preop. endo. treatment (%)							
Any	40	(34.8)	4	(17.4)	36	(39.1)	0.087
Stent	35	(30.4)	3	(13.0)	32	(34.8)	0.076
PEG^§^	4	(3.5)	1	(4.3)	3	(3.3)	1.000
EMR^¶^	1	(0.9)	1	(4.3)	0	(0.0)	0.451
Age, years (mean [SD])	63	[9]	59	[8]	64	[9]	0.015
Height, cm (median [IQR])	174	[166,179]	171	[160,174]	175	[167,180]	0.009
Preop. weight, kg (mean [SD])	74	[16]	82	[18]	73	[15]	0.017
Weight loss, kg (median [IQR])	7	[0, 13]	6	[0, 12]	8	[2, 12]	0.509
FEV1% (mean [SD])	90	[18]	90	[21]	91	[18]	0.914
Creatinine, umol/l (mean [SD])	71	[19]	72	[18]	71	[20]	0.810
CCI (median [IQR])	5	[4, 6]	5	[4, 6]	5	[5, 6]	0.098

^*^Squamous cell carcinoma

^**^Grade not reported on all pathological reports

^†^Minimally invasive esophagectomy

^‡^Video-assisted thoracoscopic surgery

^§^Percutaneous endoscopic gastrostomy

^¶^Endoscopic mucosal resection

**Fig. 2:**
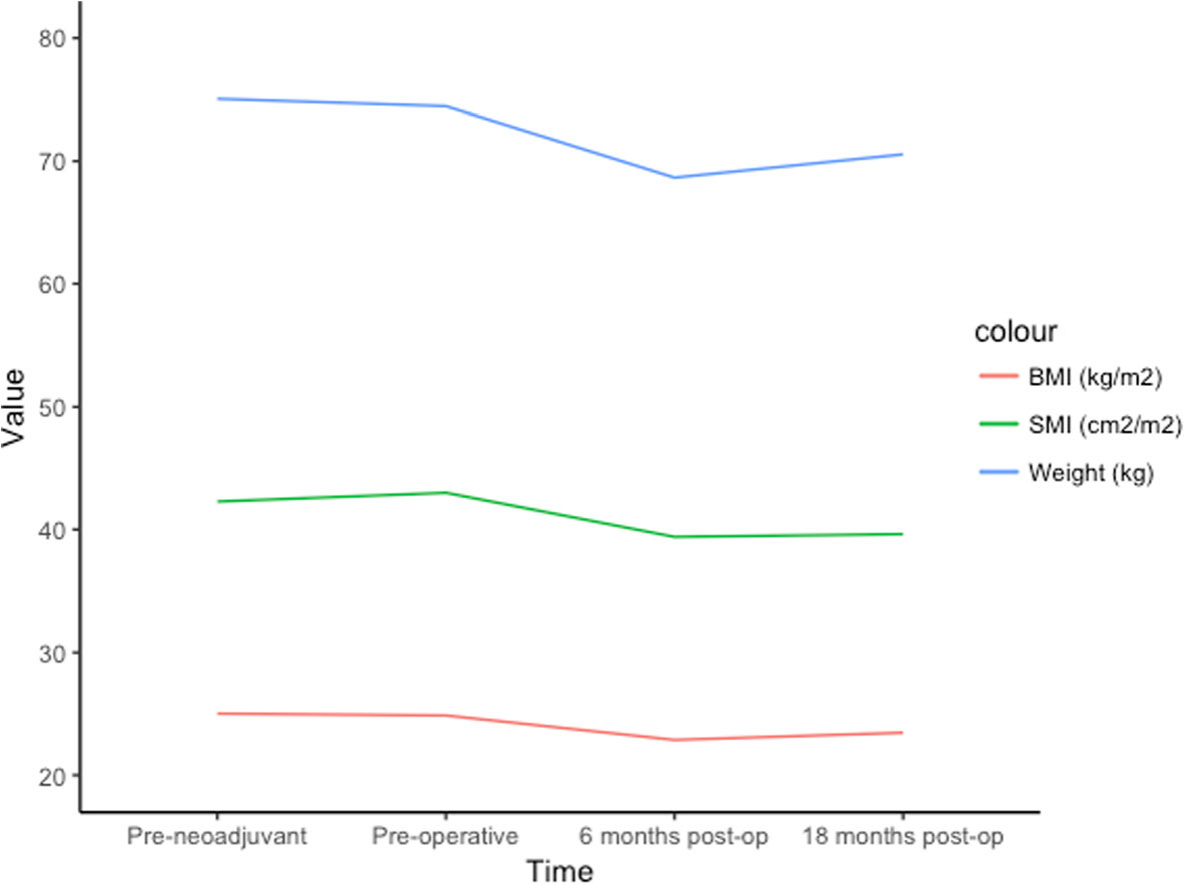
The evolution of mean body weight, body mass index, and skeletal muscle index during follow-up in the whole study population (*n* = 115)

The progression of mean weight- and body composition-related parameters during neoadjuvant treatments and follow-up is displayed in Fig. 2. Ninety-one (79.1%) patients had sarcopenia before neoadjuvant treatments, 92 (80%) were sarcopenic before esophagectomy, 82 of 99 (82.8%) were sarcopenic 6 months post-operatively, and 67 of 78 (85.9%) 18 months post-operatively.

Table 2 shows the relevant complication rates and Clavien-Dindo scores of patients. The amount of intraoperative bleeding and length of initial intensive care unit stay are also displayed. There was no statistically significant difference between the sarcopenic or non-sarcopenic groups in any complication groups. An additional table shows a more exhaustive table of complication rates [see Additional file 1]*.*

**Table 2:** Analysis of complication rates by preoperative sarcopenia

			Preoperative			
			Sarcopenia			
	Level	No		Yes		*p*
		(*N* = 23)		(*N* = 92)		
30-day mortality (%)		0	(0.0)	3	(3.3)	0.884
90-day mortality (%)		1	(4.3)	6	(6.5)	1.000
Any complication (%)		17	(73.9)	62	(67.4)	0.725
Clavien-Dindo score (%)	0	6	(26.1)	30	(32.6)	0.886
	1	0	(0.0)	3	(3.3)	
	2	4	(17.4)	10	(10.9)	
	3a	3	(13.0)	13	(14.1)	
	3b	7	(30.4)	20	(21.7)	
	4a	2	(8.7)	11	(12.0)	
	4b	0	(0.0)	2	(2.2)	
	5	1	(4.3)	3	(3.3)	
Chyle leak (%)		1	(4.3)	8	(8.7)	0.795
Anastomotic leak (%)		2	(8.7	13	(14.1)	0.729
Conduit necrosis (%)		1	(4.3)	2	(2.2)	1.000
Recurrent nerve palsy (%)		1	(4.3)	7	(7.6)	0.927
Intraoperative Complications^†^ (%)		3	(13.0)	9	(9.8)	0.939
Pulmonary complications^‡^ (%)		6	(26.1)	26	(28.3)	1.000
Reoperation rate (%)		2	(8.7)	8	(8.7)	1.000
Operative bleeding, ml (median [IQR])		200	[150, 400]	400	[150, 700]	0.091
ICU stay, days (median [IQR])		3	[1, 5]	2	[1, 4]	0.535
Tracheostomy rate (%)		1	(4.3)	8	(8.7)	0.795

^†^Intraoperative vessel, conduit or airway injury or conversion to open esophagectomy

^‡^Contains ARDS (acute respiratory distress syndrome), pneumonia, atelectasis requiring intervention, pleural effusion or pneumothorax requiring intervention, pulmonary embolism, and acute aspiration

**Fig. 3:**
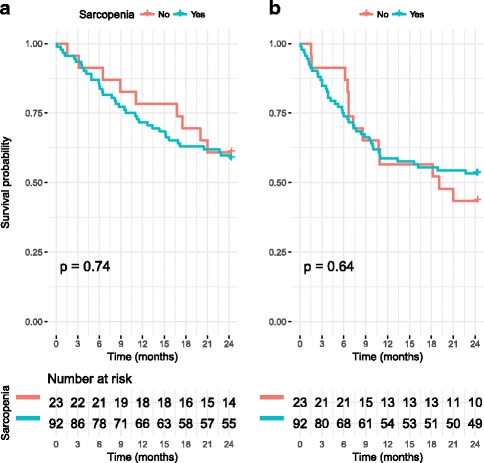
**a** Shows the 2-year Kaplan-Meier survival curve for the sarcopenic and non-sarcopenic groups and **b** shows the 2-year recurrence-free survival curves for these groups

There was no statistical difference in 2-year overall survival or recurrence-free survival between the preoperative sarcopenic and non-sarcopenic groups (*p* = 0.74 and *p* = 0.64, respectively). The Kaplan-Meier curves are shown in Fig. 3a, b. Neither preneoadjuvant SMI nor preoperative SMI had an effect on OS (*p* = 0.6023 and *p* = 0.3843) or RFS (*p* = 0.3241 and *p* = 0.9273).

**Fig. 4:**
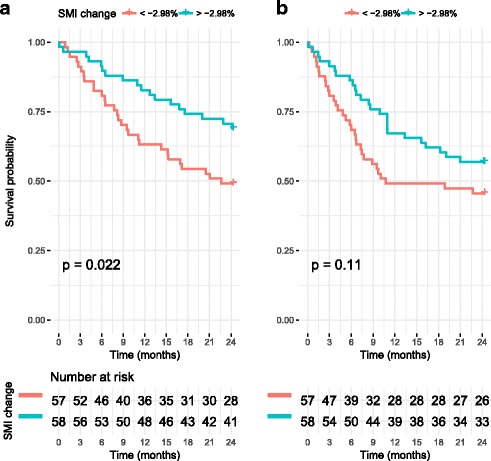
Kaplan-Meier survival analyses of the patients divided into groups by skeletal muscle index change by cut-off of − 5%. **a** Shows the 2-year overall survival rates between the groups and **b** shows the 2-year recurrence free survival

Patients were divided into groups based on the change between preneoadjuvant measurement and preoperative measurement of SMI. Median percentual change of SMI (− 2.98%) was used as the cut-off value, as has been done in a previous study [15]. The baseline characteristics are displayed in Table 3*.* The group with more muscle loss had more preoperative stents inserted (*p* = 0.037). SMI change below the median correlated with 2-year overall survival (*p* = 0.022) but not 2-year RFS (*p* = 0.11), as shown in Fig. 4a–b. The change of SMI during neoadjuvant treatments was significantly different between 2-year survivors and non-survivors with mean changes of − 0.69 and − 6.20%, respectively (*p* = 0.01259).

**Table 3:** Characteristics by SMI change groups

	SMI change				*p*
	< − 2.98%		> − 2.98%		
Number of patients (%)	57		58		
Sex (%)					
Female	19	(33.3)	10	(17.2)	0.076
Male	38	(66.7)	48	(82.8)	
ECOG (%)					
0	15	(26.3)	25	(43.9)	0.108
1	40	(70.2)	29	(50.9)	
2	2	(3.5)	3	(5.3)	
Smoking status (%)					
Current smoker	20	(35.1)	12	(20.7)	0.086
Ex-smoker	21	(36.8)	19	(32.8)	
Non-smoker	16	(28.1)	27	(46.6)	
T stage					
1	2	(3.8)	0	(0.0)	0.549
2	5	(9.3)	5	(8.6)	
3	42	(77.8)	44	(75.9)	
4	5	(9.3)	9	(15.5)	
N stage					
0	14	(25.9)	19	(32.8)	0.445
1	38	(70.4)	36	(62.1)	
2	1	(1.9)	3	(5.2)	
3	1	(1.9)	0	(0.0)	
Cancer type (%)					
Adenocarcinoma	40	(70.2)	48	(82.8)	0.111
SCC^*^	17	(29.8)	10	(17.2)	
Pathological grade**					
1	3	(13.0)	6	(18.2)	0.790
2	9	(39.1)	13	(39.4)	
3	12	(47.9)	14	(42.4)	
Tumor location (%)					
Lower third	45	(78.9)	50	(86.2)	0.357
Middle third	11	(19.3)	6	(10.3)	
Upper third	1	(1.8)	2	(3.4)	
Neoadjuvant treatment (%)					
Chemoradiation	19	(33.3)	11	(19.0)	0.079
Chemotherapy	38	(66.7)	47	(81.0)	
Operation type (%)					
MIE^†^	39	(68.4)	39	(67.2)	0.931
Thoracotomy	13	(22.8)	13	(22.4)	
Hybrid-laparoscopy	2	(3.5)	3	(5.2)	
Hybrid-VATS^‡^	2	(3.5)	1	(1.7)	
Transhiatal	1	(1.8)	2	(3.4)	
Preop. endo. treatment (%)					
Any	26	(45.6)	14	(24.1)	0.026
Stent	23	(40.4)	12	(20.7)	0.037
PEG^§^	2	(3.5)	2	(3.4)	1.000
EMR^¶^	1	(1.8)	0	(0.0)	0.993
Age, years (mean [SD])	63	[9]	63	[9]	0.817
Height, cm (median [IQR])	173	[164,179]	174	[168,179]	0.350
Preop. weight, kg (mean [SD])	74	[17]	75	[16]	0.840
Weight loss, kg (median [IQR])	10	[0, 15]	6	[2, 10]	0.131
FEV1% (mean [SD])	89	[21]	92	[16]	0.527
Creatinine, umol/l (mean [SD])	70	[22]	72	[16]	0.484
CCI (median [IQR])	5	[5, 6]	5	[4, 6]	0.397

^*^Squamous cell carcinoma

^**^Grade not reported on all pathological reports

†Minimally invasive esophagectomy

‡Video-assisted thoracoscopic surgery

§Percutaneous endoscopic gastrostomy

¶Endoscopic mucosal resection

Finally, a multivariate Cox regression analysis was done using the backwards elimination method with a *p* value limit of 0.2. The model constructed is shown in Table 4*.* The model showed that the patients who had percentual SMI change over the median (− 2.98%) had better survival than those whose SMI had decreased more (*p* = 0.049; HR − 0.609; HR 95% CI 0.297–0.997). No other covariates reached significance (N stage, T stage, or CCI). There was 11.75 events per variable (47 events, 4 variables), which is over the suggested limit of 10 (Table 4) [27].

**Table 4:** Multivariate Cox regression analysis of the covariates affecting OS

	HR	95% CI	*p*
ΔSMI%* during neoadj. (>median vs. <median)	-0.609	0.297–0.997	0.049
N stage (per level)	0.486	0.940–2.801	0.082
T stage (per level)	0.496	0.993–2.892	0.086
CCI** (per level)	0.175	0.973–1.459	0.091

^*^The percentual change in skeletal muscle index during neoadjuvant treatments

^**^Charlson comorbidity index

## Discussion

Our findings suggest that loss of skeletal muscle mass during neoadjuvant treatment of EC is a marker of poor prognosis. Sarcopenia itself was not correlated with poorer oncological outcomes; however, its prevalence is high in this population and increases post-esophagectomy.

The amount of skeletal muscle lost during neoadjuvant treatment seems to predict a poorer prognosis. A median cut-off of − 2.98% produced significantly different 2-year overall survival rates as seen in Fig. 4a. To the authors’ best knowledge, this finding has not been previously reported in published literature, although similar findings have been reported in squamous cell carcinoma patients [15]. A previous study failed to show this correlation, but concluded that the amount of skeletal muscle lost during neoadjuvant therapy differed significantly between survivors and non-survivors, which was confirmed by our study [16]. Skeletal muscle wasting post-operatively has been associated with worse outcomes in thoracic esophageal cancer [28].

The progression of sarcopenia is not stopped by resection of the tumor according to our data as shown in Fig. 2. The prevalence of sarcopenia increases with time in follow-up. Similar findings have been reported in the literature [14]. Esophagectomy seems to affect the body composition of many patients. An interventional randomized study did not see statistically significant change in the weight of patients’ post-esophagectomy at 7 days, regardless of the method of nutritional support [29]. A previous study has shown that malnutrition and weight loss is common even years after EC surgery [30]. Whether this change in body mass post-esophagectomy contributes to the morbidity or mortality of operatively treated EC patients is unknown.

Sarcopenia was not correlated with worse overall survival or recurrence-free survival in our study. This finding contradicts many previous studies and is supported by some previous studies [10–18]. Complication rates in any of the complication subgroups did not differ significantly between sarcopenic and non-sarcopenic groups. There was a statistically non-significant increase in operative bleeding with sarcopenic patients.

Our study has a number of limitations. The study is retrospective in nature without randomization. This increases the risk for systemic errors and selection bias. The number of patients included in the study is of adequate size taking into account the incidence of this disease, but statistical power is of concern especially in regard to complication rates and specific complication where the number of events is low or non-existent.

## Conclusions

In conclusion, our study found an interesting correlation between the loss of skeletal muscle during neoadjuvant treatment and worse oncological outcomes in surgically treated EC patients. This finding should guide further investigation into the interventions for nutritional support in esophageal cancer and into the significance of indirect measurements of body composition in the prognosis of EC patients.

## Additional file

Additional file 1: Complication rate analysis by preoperative sarcopenia. (XLSX 34 kb)

## Abbreviations

AC: Adenocarcinoma
BMI: Body mass index
CCI: Charlson Comorbidity Index
CI: Confidence interval
CRT: Chemoradiotherapy
CT: Computed tomography
EC: Esophageal cancer
ECOG: The Eastern Cooperative Oncology Group
EMR: Endoscopic mucosal resection
EOX: Epirubicin–oxaliplatin–capecitabine
EUS: Endoscopic ultrasound
HU: Hounsfield unit
ICU: Intensive care unit
IQR: Interquartile range
MIE: Minimally invasive esophagectomy
OS: Overall survival
PEG: Percutaneous gastrostomy
PET: Positron emission tomography
RFA: Radiofrequency ablation
RFS: Recurrence-free survival
ROI: Region of interest
SCC: Squamous cell carcinoma
SD: Standard deviation
SEMS: Self-expandable metallic stent
SMI: Skeletal muscle index
TMA: Total muscle area
VATS: Video assisted thoracoscopic surgery
